# Characterization of HA-tagged α9 and α10 nAChRs in the mouse cochlea

**DOI:** 10.1038/s41598-020-78380-5

**Published:** 2020-12-11

**Authors:** Pankhuri Vyas, Megan Beers Wood, Yuanyuan Zhang, Adam C. Goldring, Fatima-Zahra Chakir, Paul Albert Fuchs, Hakim Hiel

**Affiliations:** 1 The Center for Hearing and Balance, Department of Otolaryngology Head and Neck Surgery, Johns Hopkins University School of Medicine, 720 Rutland Avenue, Ross 818, Baltimore, MD 21205 USA; 2 Otolaryngology-Head and Neck Surgery, Renmin Hospital of Wuhan University, Wuhan, 430060 Hubei China; 3 Present Address: Sutter Instrument Company, 1 Digital Drive, Novato, CA 94949 USA

**Keywords:** Auditory system, Cellular neuroscience, Development of the nervous system, Molecular neuroscience, Peripheral nervous system, Synaptic plasticity, Synaptic transmission

## Abstract

Neurons of the medial olivary complex inhibit cochlear hair cells through the activation of α9α10-containing nicotinic acetylcholine receptors (nAChRs). Efforts to study the localization of these proteins have been hampered by the absence of reliable antibodies. To overcome this obstacle, CRISPR-Cas9 gene editing was used to generate mice in which a hemagglutinin tag (HA) was attached to the C-terminus of either α9 or α10 proteins. Immunodetection of the HA tag on either subunit in the organ of Corti of adult mice revealed immunopuncta clustered at the synaptic pole of outer hair cells. These puncta were juxtaposed to immunolabeled presynaptic efferent terminals. HA immunopuncta also occurred in inner hair cells of pre-hearing (P7) but not in adult mice. These immunolabeling patterns were similar for both homozygous and heterozygous mice. All HA-tagged genotypes had auditory brainstem responses not significantly different from those of wild type littermates. The activation of efferent neurons in heterozygous mice evoked biphasic postsynaptic currents not significantly different from those of wild type hair cells. However, efferent synaptic responses were significantly smaller and less frequent in the homozygous mice. We show that HA-tagged nAChRs introduced in the mouse by a CRISPR knock-in are regulated and expressed like the native protein, and in the heterozygous condition mediate normal synaptic function. The animals thus generated have clear advantages for localization studies.

## Introduction

The mammalian cochlea is subject to modulatory feedback that arises near the superior olivary complex in the brainstem^1^. Lateral olivocochlear (LOC) efferents form synapses with radial afferent fibers underneath inner hair cells (IHCs), while medial olivocochlear (MOC) efferents establish synapses with immature IHCs, and exclusively with outer hair cells (OHCs) in the adult. Both unmyelinated LOC and myelinated MOC fibers express cholinergic markers^2^, and acetylcholine mediates the known effects of efferent transmission onto vertebrate hair cells^3–13^. Upon its release, acetylcholine activates a nicotinic acetylcholine receptor (nAChR) composed of α9 and α10 subunits^14–17^. In vertebrates the expression of these 2 subunits is limited mainly to the cochlear and vestibular end organs^14,15,18,19^. Previous mRNA in situ hybridization studies implicated α9 and α10 containing receptors in other physiological roles such as inflammatory immune responses^20–22^ and nociception^23,24^. It has been difficult to confirm these results at the level of protein expression or by functional tests. Reliable antibodies have been elusive and those employed diverged from their respective mRNA expression in the inner ear epithelia and other organs^25–28^. Establishing a reliable method for visualizing α9 and α10 containing nAChRs will advance our knowledge about their role in different cellular systems.

CRISPR-Cas9 technology (Clustered Regularly Interspaced Short Palindromic Repeats, Caspase 9) provides an efficient means to knock-in a peptide tag sequence within a protein of interest. Here the CRISPR-Cas9 approach was used to attach a 9 amino acid hemagglutinin (HA) tag to the C-terminus of α9 and α10 subunits^29,30^. The wide availability of antibodies against peptide tags such as the HA epitope provides visualization of specific proteins in biological tissues, while providing a robust negative control in the form of wild type tissue. In the present study, the expression of α9 and α10 subunits was visualized in the prehearing and adult cochlear sensory epithelium. In both homozygote and heterozygote HA-tagged mice, the α9 and α10 subunits cluster in the hair cell membrane immediately facing the efferent boutons. The cochlear expression of α10HA protein is comparable to that of α9HA in both neonatal and adult animals. Immunopuncta for α9HA or α10HA in the prehearing cochlea were more numerous and smaller in size than those observed in the adult cochlea. Hearing thresholds were normal in heterozygous and homozygous HA-tagged mice. Hair cells expressing either α9 or α10 HA-tagged subunits responded to ACh released by electrical stimulation of cochlear efferents. The postsynaptic currents had inward and outward components, consistent with activation of nAChRs and associated calcium-dependent potassium channels. Recordings from α9HA or α10HA heterozygote hair cells were similar to those of wild type hair cells in amplitude and time course. Synaptic responses in the homozygous HA-tagged IHCs were smaller and occurred with lower probability. The present work describes HA tagged animal models for α9 and α10 nAChRs that will facilitate efforts to explore further the role of the efferent system in cochlear development, aging and after acoustic trauma.

## Results

### Generation of HA tagged α9 and α10 mice using CRISPR/Cas9 technology

**Figure 1:**
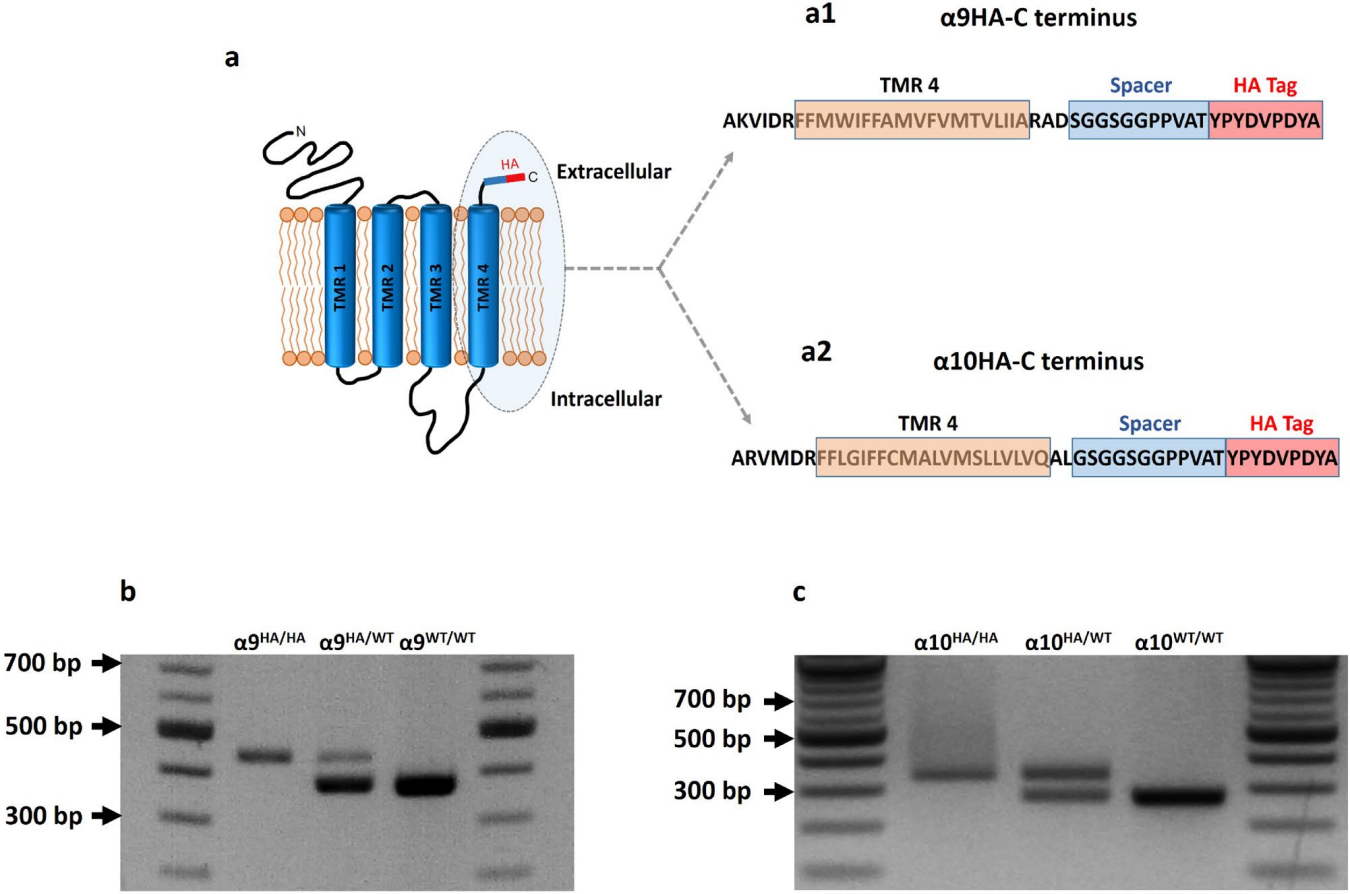
Schematic of α9 and α10 nAChR topology in membrane bilayer (**a**) with an enhancement of the shaded ellipse of the C-terminus of α9 (**a1**) and α10 (**a2**). Amino acid sequence depicting part of the intracellular loop, the transmembrane region 4 (TMR 4) and the extracellular amino acids attached to the HA tag complex (Spacer blue box + HA red box). The gel images show predicted PCR products for WT mice and HA tagged α9 (**b**) and α10 (**c**) HA/HA and HA/WT mice. The predicted sizes are 435 bp α9^HA/HA^, 375 bp WT/WT, 334 bp α10^HA/HA^ and 271 bp for WT/WT.

Alpha9 and α10 nAChR proteins display 50–60% structural homology and both have four transmembrane regions (TMRs). The C-terminal extracellular domain of α9 and α10 nAChRs exhibits several extracellular amino acids, where a spacer and a nine amino acid hemagglutinin peptide (HA) were introduced immediately after the fourth transmembrane region (TMR4) (Fig. 1a,a1,a2).

The single strand DNA (ssDNA) repair template used to incorporate the HA coding sequence and the guide RNA along with individual sequences for PCR screening primers are shown in Supplementary Table 1. The protospacer adjacent motif (PAM) is underlined in the ssDNA construct. The expected PCR amplicons from screening of WT, homozygous (HA/HA) and heterozygous (HA/WT) α9 and α10 mice are shown in Fig. 1b,c, respectively, and Supplementary Fig. 1.

Alpha9 nAChR subunit tagging yielded thirty-two founder mice, among which six were found to have the correct in-frame insertion of the HA tag without any undesirable deletions or mutations. The alpha10 cohort yielded fifty-two founder mice, out of which four had the appropriate in-frame insertion. The selected mice were bred with FVB.129P2 mice (to avoid the early onset hearing loss that afflicts C57BL6 mice), and their offspring were genotyped and sequenced for two generations The mice were bred as homozygous or heterozygous colonies for the purpose of testing for differences between the two genotypes. Sequence analysis confirmed the presence of the HA tag for two generations, showing the faithful germline transmission of the inserted tag to subsequent generations.

### Auditory brainstem responses (ABRs) of α9HA α10HA mice

**Figure 2:**
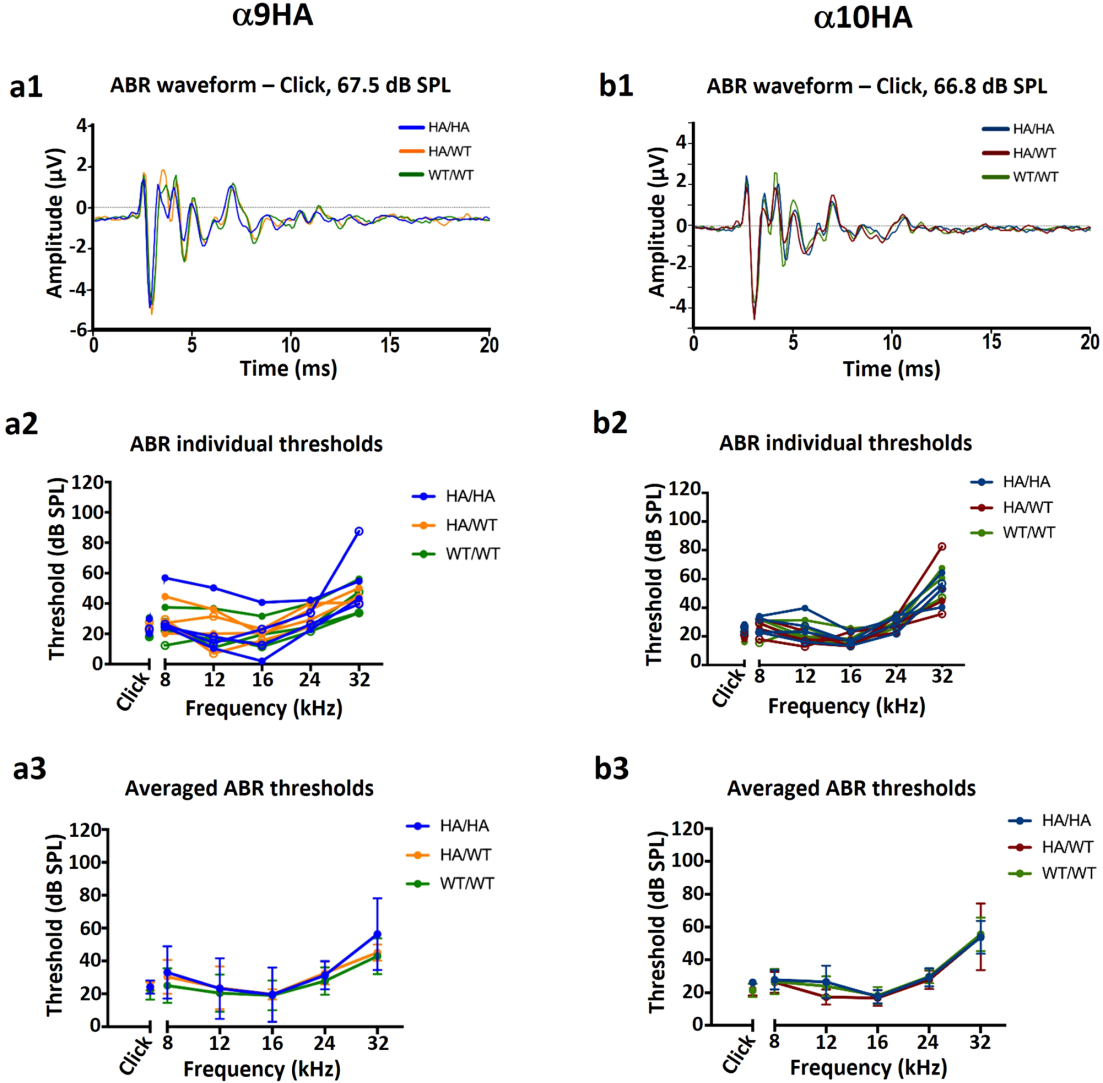
Alpha9HA and α10HA knock-in mice exhibit normal ABRs to both click and pure tone stimuli. Alpha9HA ABR waveform (**a1**), individual threshold measurements (**a2**), and average threshold measurements by genotype group (**a3**). The overlaid ABR waveforms are the average waveforms generated from the click stimulus at 30 dB of attenuation from the maximum stimulus tested, as indicated. Each colored line is the average of 4 mice in that genotype group. N = 4 mice in each group. Averaged thresholds (bottom) were not significantly different among groups when tested with a two-way ANOVA comparing the three genotypes (F(2,53) = 1.15, *p* = 0.32). Dunnett’s correction for multiple comparisons was applied to the comparisons between each genotype at each frequency; no genotype was significantly different from any other genotype at each frequency. Alpha10HA ABR waveform (**b1**), individual threshold measurements (**b2**), and averaged threshold measurements by genotype group (**b3**). The overlaid ABR waveforms are the average waveforms generated from the click stimulus at 30 dB of attenuation from the maximum stimulus tested, as indicated. Each colored line is the average of 4 mice in that genotype group. N = 4 mice in each group. Averaged thresholds (bottom) were not significantly different among groups when tested with a two-way ANOVA comparing the three genotypes (F(2,53) = 0.8, *p* = 0.45). Dunnett’s correction for multiple comparisons was applied to the comparisons between each genotype at each frequency; no genotype was significantly different from any other genotype at each frequency.

ABR threshold measurements were made from wildtype (WT/WT, N = 4), heterozygote (HA/WT, N = 4), and homozygote (HA/HA, N = 4) α9HA (Fig. 2a) and α10HA (Fig. 2b) mice. The wildtype animals used in the ABR experiments were offspring of heterozygous parents ensuring they were an appropriate background strain control. Each group included even numbers of male and female mice to exclude variation based on sex. The α9HA cohort of mice (Fig. 2a1–a3) was 2 months old, the α10HA cohort was 1.5 to 2.5 months old (Fig. 2b1–b3). The overlaid waveforms of WT, heterozygous, and homozygous mice with the α9HA and α10HA mutations showed no difference in latency or amplitude compared to WT/WT (Fig. 2a1,b1). The average wave 1 amplitudes for α9HA cohort at 67.5 dB SPL were 6.5 ± 1.5 µV (WT/WT), 7.7 ± 2.3 µV (HA/WT), and 6.3 ± 2.9 µV (HA/HA) (Fig. 2a1). Wave 1 amplitude was not significantly different from the wildtype (F(2,63) = 1.86, *p* = 0.16; adjusted *p* value for WT/WT vs α9HA/HA = 0.71; adjusted *p* value for WT/WT vs α9HA/WT = 0.11) (Supplementary Fig. 2a). Latency to the peak of wave 1 was not significantly altered when either mutant group was compared to wildtype (HA/HA vs WT/WT adjusted *p* value = 0.15; HA/WT vs WT/WT adjusted *p* value = 0.058.) (Supplementary Fig. 2b). While the individual mice from the α9HA cohort showed some variation in ABR thresholds, the averaged thresholds from WT compared to heterozygous or homozygous animals were not significantly different (F(2,53) = 1.15, *p* = 0.32; adjusted *p* value for WT/WT vs HA/HA = 0.24; adjusted *p* value for WT/WT vs HA/WT = 0.56) (Fig. 2a1–a3). The α10HA cohort had consistent waveforms and thresholds for all groups (Fig. 2b1–b3). The averaged thresholds for each mutant α10HA genotype were not significantly different from the wildtype thresholds (F(2,53) = 0.8, *p* = 0.45; adjusted *p* value for WT/WT vs HA/HA = 0.89; adjusted *p* value for WT/WT vs HA/WT = 0.61). The average wave 1 amplitudes at 66.8 dB SPL were 6.4 ± 1.1 µV (WT/WT), 6.9 ± 1.5 µV (HA/WT), and 7.3 ± 2.0 µV (HA/HA) (Fig. 2b1). Wave 1 amplitude was not significantly different from the wildtype (F(2,63) = 1.53, *p* = 0.22; adjusted *p* value for WT/WT vs α10HA/HA = 0.15; adjusted *p* value for WT/WT vs α10HA/WT = 0.59) (Supplementary Fig. 2c). Latency to the peak of wave one was not significant when either mutant group was compared to wildtype (HA/HA vs WT/WT adjusted *p* value = 0.054; HA/WT vs WT/WT adjusted *p* value = 0.98.) (Supplementary Fig. 2d). A larger degree of variation of ABR threshold was observed in α9HA cohort mice, but it falls within the hearing threshold range for WT mice. The α10HA cohort of mice is on the same background and did not exhibit the same variability in ABR thresholds. These experiments show that heterozygous and homozygous mice with HA tags on either α9 or α10 appear to respond equally with wildtype mice when presented with click and pure-tone stimuli.

### Validation of HA tag expression in the cochlea

**Figure 3:**
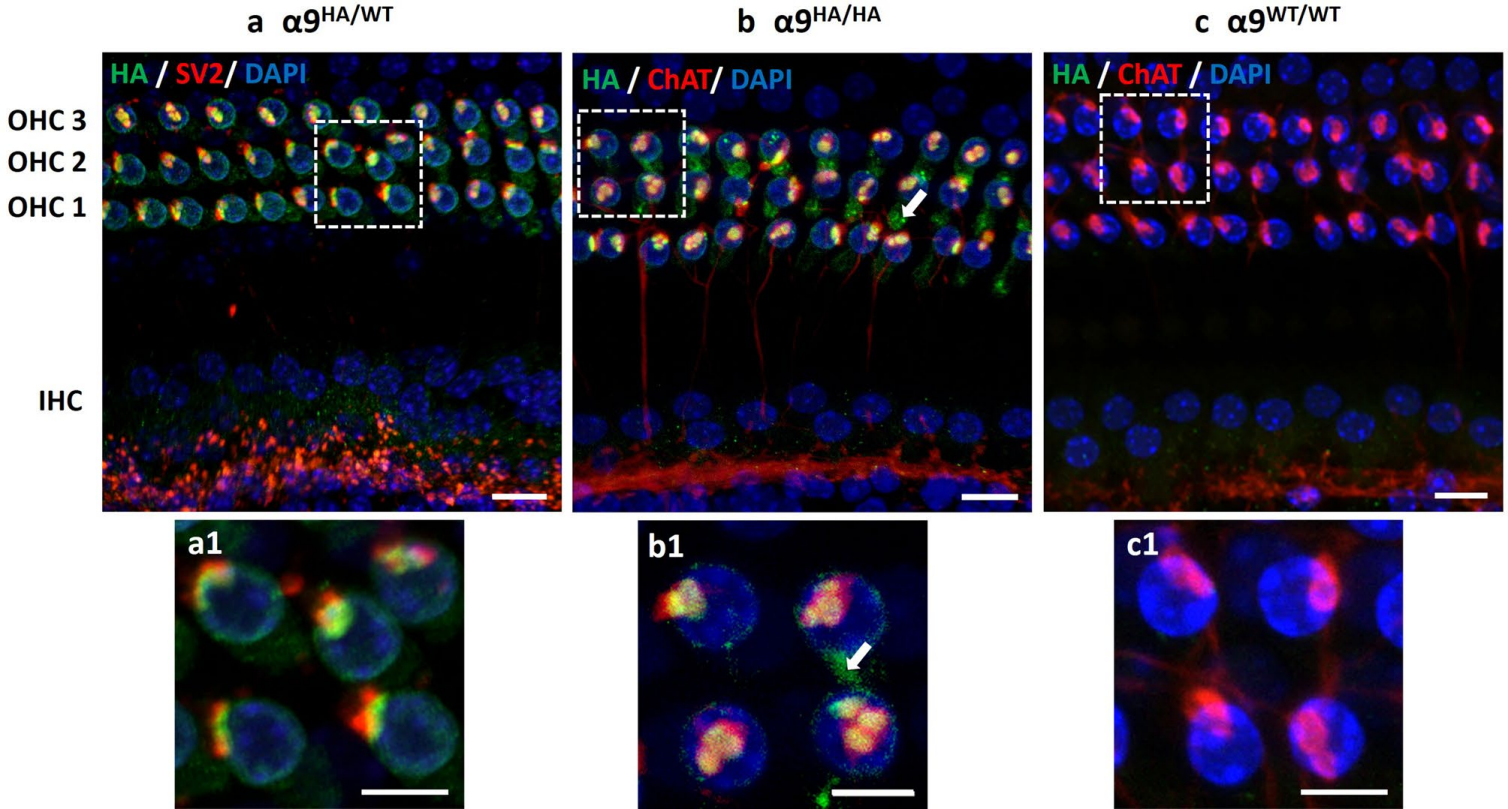
Immunolabeling of MOC terminals and hemagglutinin tag (HA) in the cochlea of adult HA-tagged and wild type mice (P21–P60). (**a**) Maximum intensity projection images from the midturn of organ of Corti probed with anti-HA tag (green) and anti-SV2 (red) in α9^HA/WT^ (**a**) or anti-ChAT antibodies (red) in α9^HA/HA^ (**b**) and α9^WT/WT^ (**c**) show the MOC terminals (red) juxtaposed to immunosignal clusters for HA tag. Note a clear absence of immunolabeling for HA tag in WT cochlea (**c**). Nuclei are visualized by DAPI staining (blue). Subcuticular immunopuncta in OHCs is indicated by arrows (**b**, **b1**). Insets represent magnified areas marked by squares in images (**a**)–(**c**). Scale bar: (**a**)–(**c**) 10 µm, insets (**a1**)–(**c1**) 5 µm. (α9^WT/WT^ N = 5, α9^HA/HA^ N = 16, α9^HA/WT^ N = 9).

The localization of HA-tagged α9 nAChRs was examined in the cochleas of adult (postnatal day (P) 21–60) heterozygous (α9^HA/WT^) and homozygous (α9^HA/HA^) mice and their wild-type WT/WT) littermates. In both α9^HA/WT^ (Fig. 3a) and α9^HA/HA^ cochleas (Fig. 3b), the HA tag formed immunopuncta clustered at the synaptic pole of all three rows of OHCs; consistent with postsynaptic surface membrane receptor amassed at the synaptic contacts and reminiscent of AChR labeling at the neuro-muscular junction^31^. Furthermore, these clusters of HA immunopuncta were absent from the IHC area where cholinergic LOC neurons make synaptic contacts with type I afferent dendrites (mediated presumably by other AChRs). The immunoclusters were observed only at the base of OHCs and were juxtaposed and coextensive with synaptic vesicle 2 (SV2) or choline acetyl transferase (ChAT) positive MOC efferent terminals. In the wild type littermate control mice, the cochlear partition was devoid of any HA immunolabel (Fig. 3c), while MOC terminals were brightly positive for ChAT protein. The pattern of HA tag immunolabeling was consistent in both heterozygote and homozygote mutant mice with no obvious differences in synaptic arrangements.

### Expression of HA-tagged α9 and α10 nAChRs in the adult cochlea

**Figure 4:**
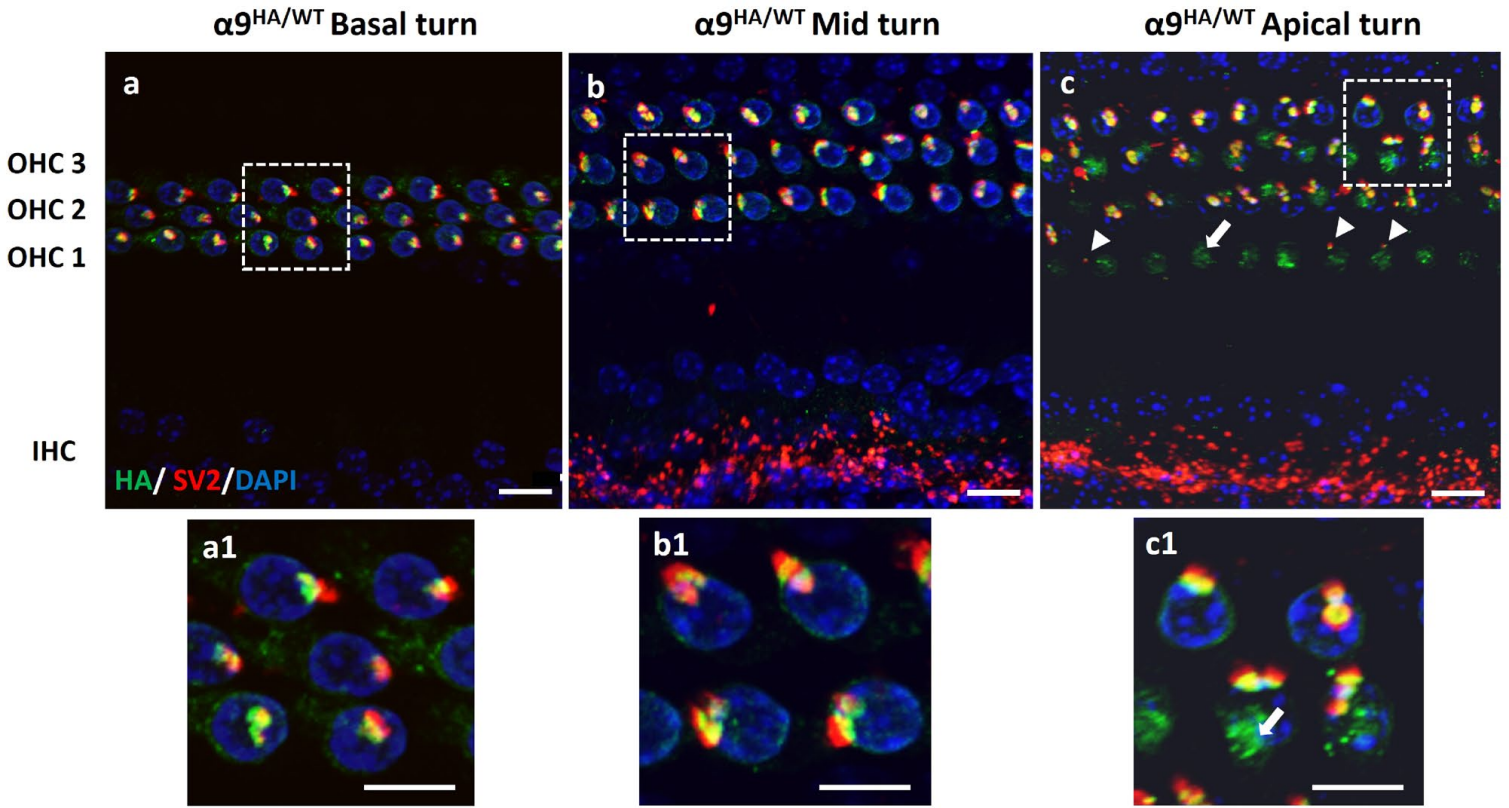
Expression of α9 nAChR subunit in α9^HA/WT^ adult mouse (P21–P60). Co-immunostaining of organ of Corti with MOC terminal marker (SV2, red) and anti-HA tag antibodies (green). Along the sensory epithelium from basal to apical turn, OHCs exhibited postsynaptic α9HA immunoclusters (green) at their sub-nuclear pole region. These immunoclusters are juxtaposed to the SV2-positive MOC boutons (red) throughout the sensory epithelium. In panel 4c, arrowheads indicate the presence of efferent contacts near the cuticular plate juxtaposed to α9 immunopuncta. Subcuticular immunopuncta in OHCs are indicated by arrows (**c**, **c1**). Insets represent magnified areas marked by squares in images (**a**)–(**c**). The maximum intensity projection image stack in 4a was outside the range of IHC, hence LOC efferents and IHCs are not visible. Nuclei are visualized by DAPI staining (blue). Scale bar: (**a**)–(**c**) 10 µm, insets (**a1**)–(**c1**) 5 µm. (α9^HA/WT^ N = 9).

**Figure 5:**
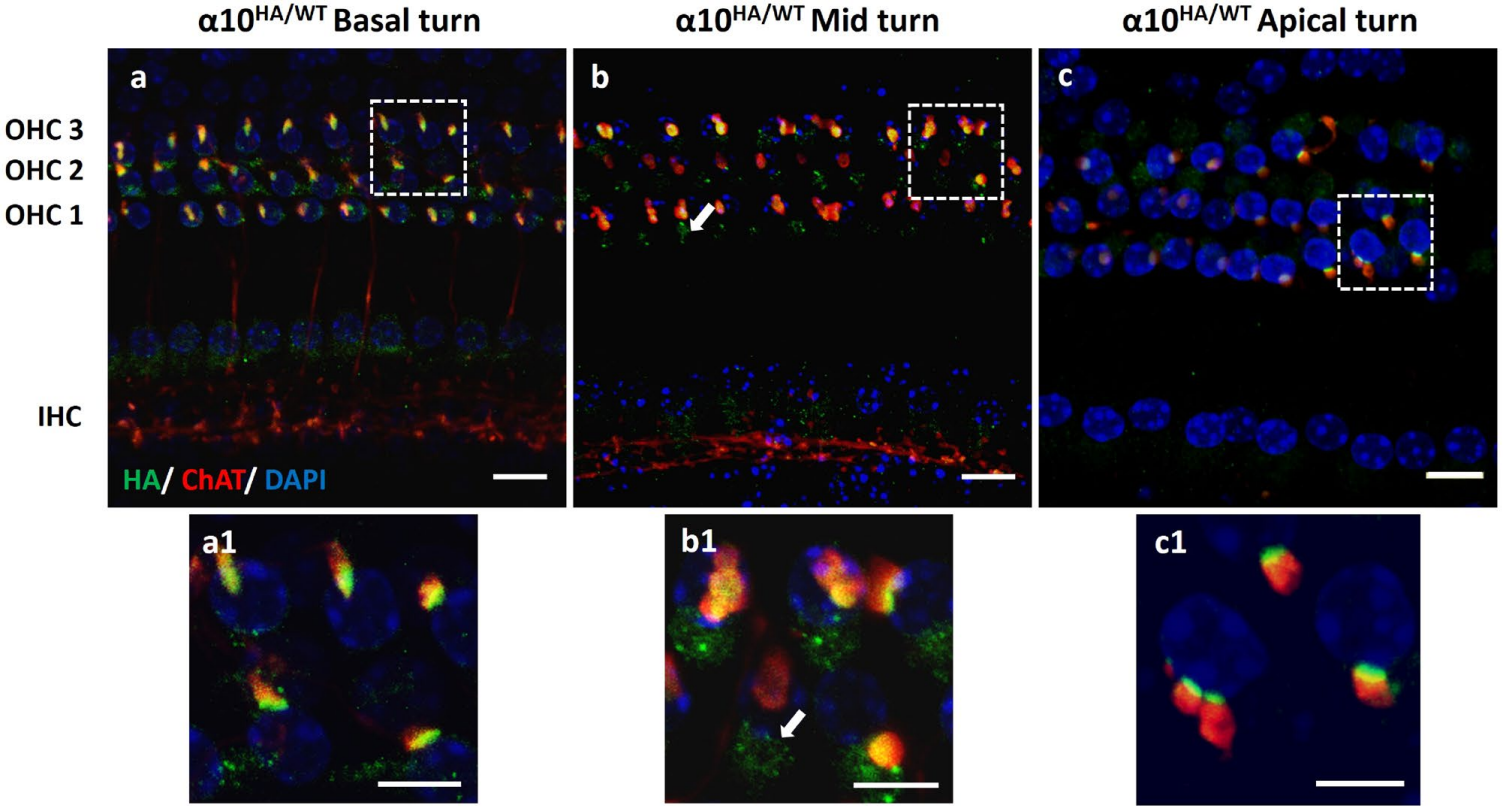
Expression of α10 nAChR subunit in α10^HA/WT^ adult mouse (P21–P60). Maximum intensity projection images show immunolabeling for MOC terminals (ChAT, red) and HA tagged α10 subunit (green) along the cochlear tonotopic axis. Alpha10HA immunoclusters were juxtaposed and coextensive with ChAT positive efferent terminals beneath the 3 rows of OHCs. Insets represent magnified areas marked by squares in images (**a**)–(**c**). Subcuticular immunopuncta in OHCs are indicated by arrows (**b**, **b1**). The maximum intensity projection image stack in 5c was outside the range of the lateral efferent system. Nuclei are visualized by DAPI staining (blue). Scale bar: (**a**)–(**c**) 10 µm, (**a1**)–(**c1**) 5 µm. (α10^HA/WT^ N = 15).

The distribution of HA tag along the tonotopic axis was evaluated in both α9HA and α10HA mouse lines using immunohistochemistry to label the HA tag epitope and for biomarkers of MOC terminals (SV2 or ChAT). The expression pattern of α9^HA/WT^ mice is shown at the cochlear basal, mid and apical turn (Fig. 4a–c) of adult mice (P21–60). Immunoclusters at the base of OHCs were juxtaposed to the SV2-positive MOC terminals throughout the base-apex axis. Adjacent SV2-positive MOC terminals aligned with a single, wide α9HA immunocluster, or could occur as spatially distinct MOC terminals juxtaposed to separate postsynaptic, HA immunopunta on OHCs (Fig. 4 insets a1, b1 and c1). Often OHCs showed bright immunopuncta in the cytoplasm, particularly in the Golgi-rich subcuticular region (Figs. 3b, 4c and 5b, arrows). This is particularly noticeable in the apical turn example of α9HA immunostaining (Fig. 4c). In these samples, the organ of Corti pressed by the cover glass tilted the hair cell bodies so that the synaptic pole of an OHC from one row is in the same focal plane as the cuticular plate area of the OHCs from the neighboring row. Efferent boutons associated with HA immunopuncta in the baso-lateral area of the OHC just below the cuticular plate were observed occasionally, as depicted in Fig. 4c (arrowheads). Efferent synapses near the cuticular pole of OHCs of various species have been reported previously^32–35^.

Alpha10HA protein distribution was similar to that of α9HA protein throughout the cochlear duct. Alpha10HA protein immunoclusters were only present at the synaptic zone of the three rows of OHCs and juxtaposed with ChAT-positive MOC terminals (Fig. 5a–c, insets a1–c1).

Efferent synapses were universally associated with HA immunopuncta. Quantitative analysis of efferent contacts per OHC was performed for adult WT, α9HA and α10HA mice. No significant differences were observed between the different genotypes. The number of efferent boutons per OHC was 2.41 ± 0.34 for WT, 2.43 ± 0.45 for α9^HA/WT^, 2.12 ± 0.26 for α9^HA/HA^, 2.38 ± 0.26 for α10^HA/WT^ and 2.21 ± 0.44 for α10^HA/HA^ mice (Supplementary Fig. 3).

Together these observations show that both α9HA and α10HA genes are being transcribed, their mRNAs translated, and their proteins inserted into the plasma membrane in the postsynaptic membrane of OHCs.

### Expression of HA-tagged α9 and α10 nAChRs in pre-hearing mice

**Figure 6:**
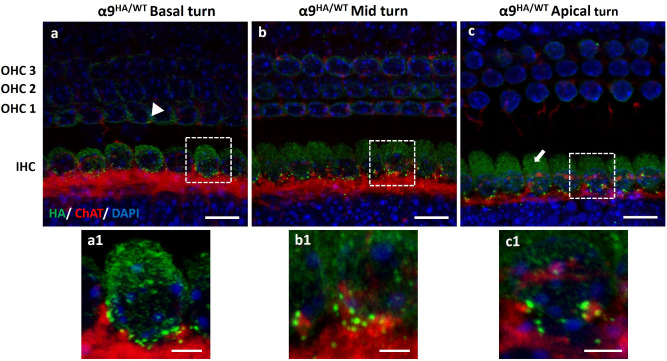
Expression of α9HA subunit in the developing cochlear sensory epithelium (P7–P8). Immunostaining of MOC axons with anti-ChAT antibody (red) and α9HA with anti-HA antibody (green). Insets (**a1**–**c1**) represent the magnification of a single IHC synaptic zone from each region of the cochlea represented above (**a–c**). Occasionally, α9HA immunopuncta are seen in the OHCs area, primarily in the first row (arrowhead, **a**). Diffuse cytosolic labeling in IHCs is indicated by arrow (**c**). Nuclei are visualized by DAPI staining (blue). Scale bar: (**a**)–(**c**) 10 µm, (**a**)–(**c1**) 2.5 µm. (α9^HA/WT^ N = 8).

**Figure 7:**
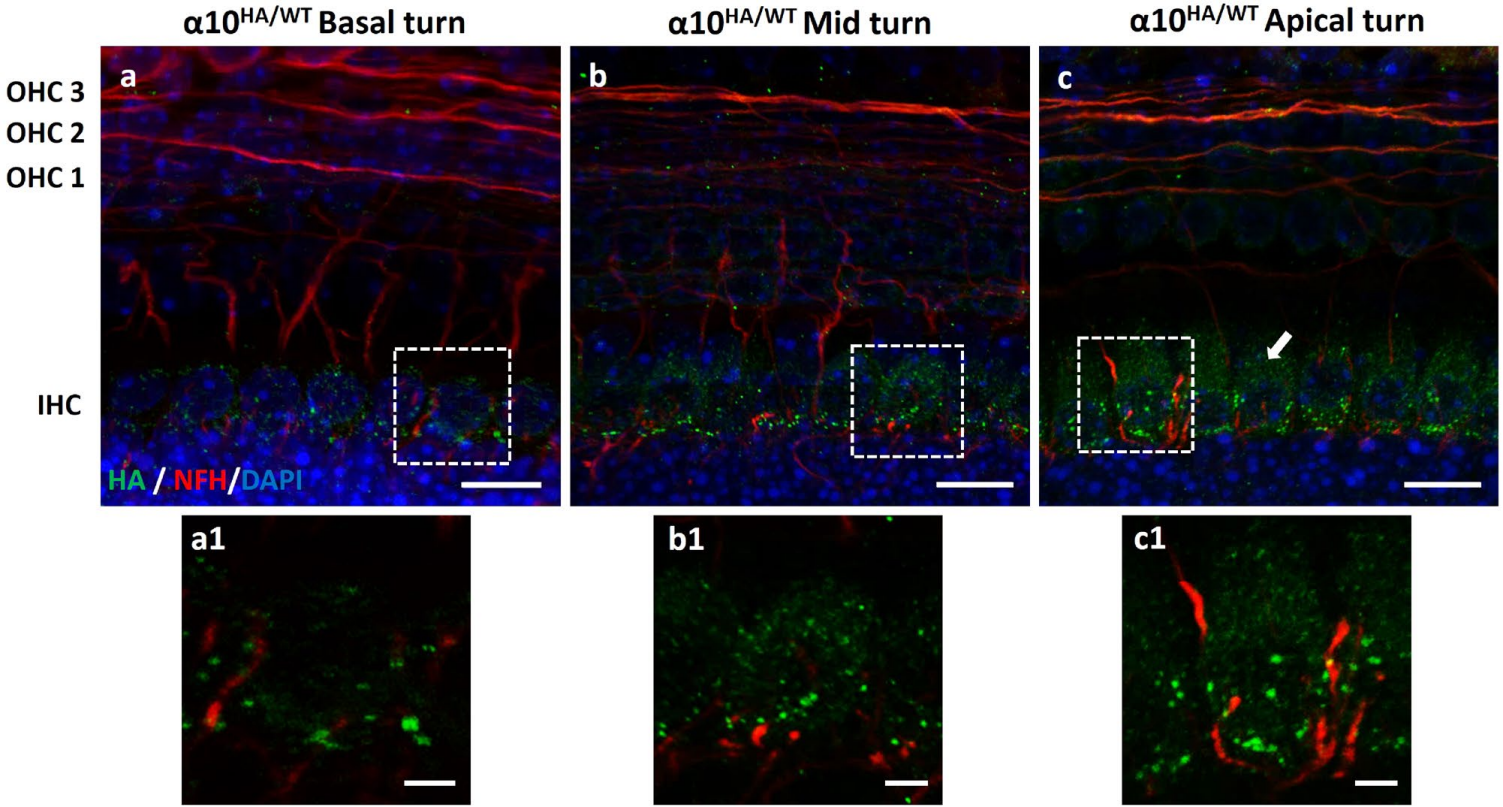
Expression of α10HA subunit in the developing cochlear sensory epithelium (P7–P8). The distribution of α10HA protein along the cochlea reflected that of its analogous α9HA subunit, suggesting a close structural and functional association (**a**–**c**). Similarly, α10HA protein immunopuncta were small, abundant, and arrayed around IHCs synaptic pole. Adjunct images (**a1**–**c1**) are a magnification of IHC synaptic pole detailing the immunolabeling of α10HA subunit surface membrane proteins. Diffuse cytosolic labeling in IHCs is indicated by arrow (**c**, **c1**). Nuclei are visualized by DAPI staining (blue). Scale Bar: (**a**–**c**) 10 µm, (**a1**–**c1**) 2.5 µm. (α10^HA/WT^ N = 10).

It is well established that prior to the onset of hearing, olivocochlear efferents transiently form synapses on IHCs^36–38^. By the onset of hearing (P12–P14), the MOC axons synapse with OHCs and contacts on IHCs are lost. These histological observations were corroborated by several electrophysiological reports^39–44^. These studies indicated that MOC fibers make transient, hyperpolarizing, cholinergic synapses onto IHCs and inhibit spontaneous calcium action potentials generated in IHCs before the onset of hearing. Hence, the expression of both α9HA and α10HA in the developing sensory epithelium was examined in P7–P8 neonatal mutant mice (Figs. 6 and 7, respectively). To visualize the distribution of α9 and α10 nAChRs, cochleas from prehearing mice were examined by co-immunolabeling the HA tag and ChAT or neurofilament heavy chain (NFH). The expression of α9HA and α10HA proteins was mainly limited to IHCs and was distributed along all the cochlear turns (Figs. 6a–c, 7a–c and insets). Alpha9HA and α10HA immunolabeling was mainly at the basal pole of IHCs and formed a ring of immunopuncta around the cell body (insets Figs. 6a1–c1, 7a1–c1). In addition to these bright synaptic immunopuncta, IHCs displayed diffuse cytoplasmic label (arrows, Figs. 6c, 7c). The α9HA or α10HA immunopuncta were associated with a plexus of ChAT-positive fibers passing beneath IHCs. This is in contrast to the adult OHCs where each MOC terminal is dedicated to a single α9HA or α10HA immunopunctum. Also, occasional α9HA immunopuncta are seen in the OHC area, primarily in the first row (Fig. 6a, arrowhead). Consistent with ultrastructure studies reporting many small efferent contacts on IHCs^45–47^, HA tag immunopuncta in neonatal IHCs were smaller and more abundant than those found in the adult OHCs.

HA tag immunolabeling in α9HA and α10HA mutant mouse lines showed similar expression patterns during development and throughout the cochlear sensory epithelium. The shape and postsynaptic positioning of HA tag immunolabeling shows that the distribution of HA tag reflects expression of either α9 or α10 in the cochlea, and is consistent with previous physiological reports that established the distribution and function of α9α10 nAChRs in vertebrate OHCs^7,8,11,12,14,15,35^. These results demonstrate that HA tagging did not alter expression or localization of the protein. The diffuse cytosolic labeling of some samples may be due to accumulation of the HA tag in endoplasmic compartments.

Previous heterologous expression in oocytes, as well as physiological and pharmacological evidence from vertebrate sensory hair cells, indicated that α9 and α10 subunits assemble to form functional nAChRs^15–17^. The close correspondence of expression patterns for HA-tagged α9 or α10 proteins supports the conclusion that these subunits combine to form the developmental as well as adult nAChRs of IHCs and OHCs, respectively.

### Synaptic function of HA-tagged α9 and α10 nAChRs

**Figure 8:**
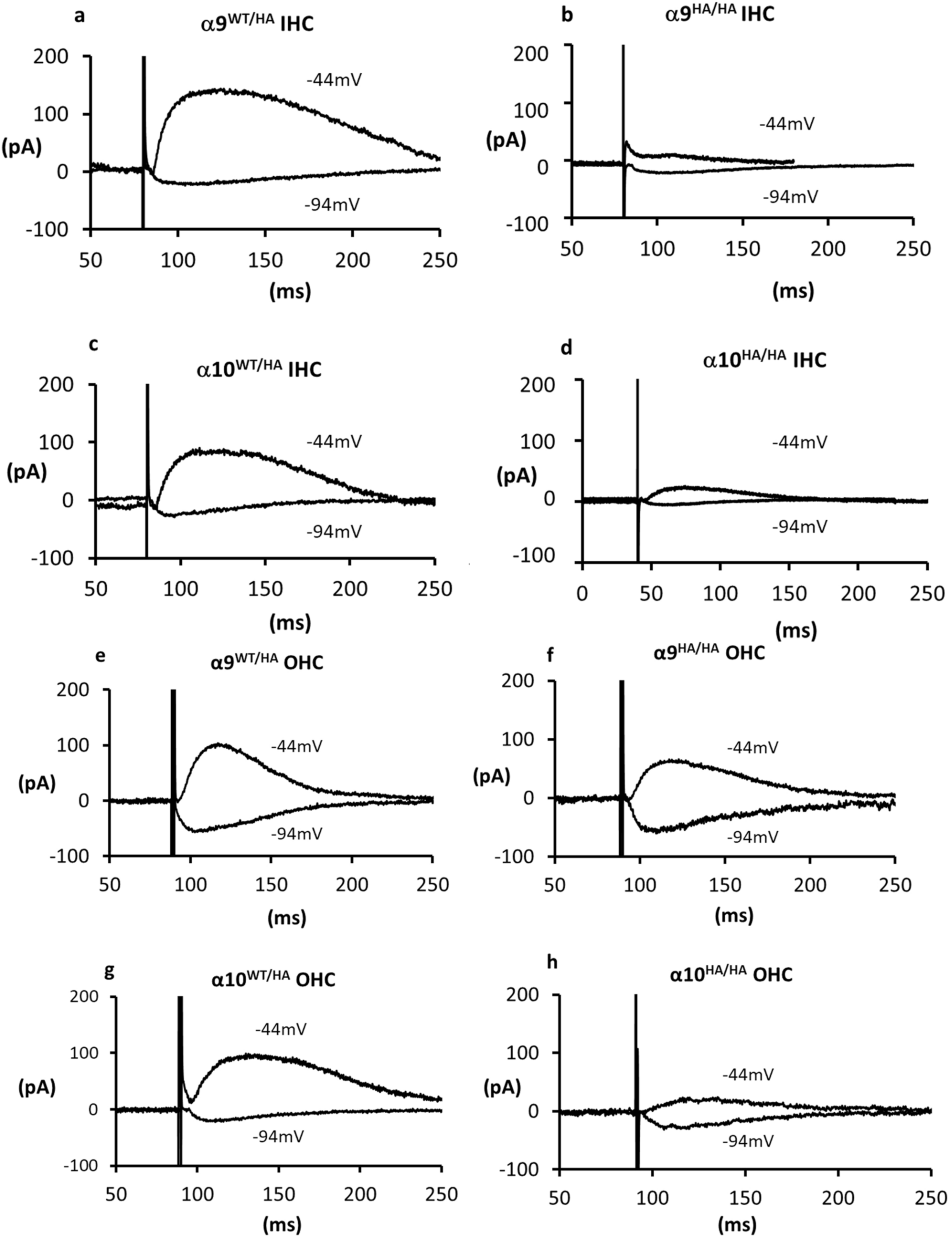
Efferent synaptic currents in inner hair cells (IHCs) and outer hair cells (OHCs). Average waveform of successes during 1 Hz stimulus train (vertical line is the shock artifact). Outward current and inward current from IHCs are shown here for different genotypes—α9^HA/WT^ (**a**), α9^HA/HA^ (**b**), α10^HA/WT^ (**c**) and α10^HA/HA^ (**d**). Outward current and inward current from OHCs in different genotypes are shown here—α9^HA/WT^ (**e**), α9^HA/HA^ (**f**), α10^HA/WT^ (**g**) and α10^HA/HA^ (**h**). Hair cell membrane potentials are indicated for each recording. Baseline subtracted from all records for direct comparison of waveform. All recordings were performed at postnatal ages P9–P11.

Effective utilization of HA-tagged nAChR mice depends on knowing whether synaptic function has been altered by this modification. This was examined at the cellular level using intracellular voltage-clamp recording from IHCs and OHCs in apical cochlear tissue excised from young heterozygous and homozygous mice (P9–P11). Initial experiments involved application of ACh or stimulation of synaptic release by depolarizing efferent contacts using high potassium saline. Membrane currents could be evoked in HA-tagged inner and outer hair cells by these techniques. However, ‘puffer’ application of ACh depended critically on positioning in the intact tissue and so was quite variable from trial to trial. Synaptic currents in 40 mM potassium saline were entirely inward, obscuring the possible contribution of potassium channels to the postsynaptic response, and revealed little about presynaptic function. Thus, it proved more informative to evoke synaptic release with electrical shocks, providing quantification not only of the compound postsynaptic response, but also an estimate of presynaptic release efficacy. Depending on membrane potential, inward or outward postsynaptic currents could be evoked in IHCs and OHCs from all four genotypes. Exemplar postsynaptic currents resulting from electrical stimulation of efferent axons are shown for young IHCs from both heterozygous and homozygous α9HA and α10HA mice (Fig. 8a–d). Negative to the potassium equilibrium potential (E_K,_ − 81 mV) membrane current was entirely inward. Positive to E_K_, membrane current was outward, consistent with the known involvement of calcium-gated potassium channels in the cholinergic response. These records are the average of evoked responses (not including failures or multi-peaked responses) during trains of 50 or 100 shocks at 1 or 5 Hz. The amplitudes and time constants of decay of postsynaptic currents in the heterozygote IHCs were not statistically different from those of IHCs from wildtype littermates (Supplementary Tables 2 and 3) and were comparable to those reported previously for mouse inner and outer hair cells^48–54^.

However, postsynaptic amplitude was significantly smaller in the homozygous α9^HA/HA^ IHCs than in IHCs from the heterozygous α9^HA/WT^ mice (Supplementary Tables 2 and 3). Also, the response probability (fraction of successes) was significantly lower onto α9^HA/HA^ IHCs than onto the heterozygous IHCs. The quantum content (fraction of failures method) differed approximately three-fold (1.27 for α9^HA/WT^ vs 0.46 for α9^HA/HA^).

Inward and outward synaptic currents also were obtained from α10^HA/WT^ and α10^HA/HA^ IHCs (Fig. 8c,d). As for the HA-tagged α9 mice, response amplitude was statistically significantly larger in heterozygous than in homozygous IHCs (Supplementary Tables 2 and 3). Synaptic responses were very infrequent in the α10^HA/HA^ IHCs and so release probability was increased by measurement in 5 mM external calcium. Under these conditions the response probability was near to that for α10^HA/WT^ recorded in 1.3 mM calcium (quantum content 0.99 for α10^HA/WT^ vs. 0.78 for α10^HA/HA^).

Efferent synaptic recordings from OHCs were rarer in these tissues from young mice, presumably due to the delayed innervation of OHCs compared to IHCs^43^. Consistent with previous reports from wild type mice^48^, even when synaptically active, response probability was significantly lower onto OHCs than IHCs for each genotype (Supplementary Table 4), even though OHC recordings were conducted with elevated external calcium (5 mM versus 1.3 mM for most IHC recordings). Nonetheless, a similar pattern emerged; inward and outward synaptic currents could be obtained from OHCs of all genotypes (Fig. 8e–h). In contrast to the case for IHCs, average response amplitude did not differ significantly between heterozygote and homozygote OHCs. Response probability was significantly lower only for α9^HA/HA^ OHCs compared to that for α9^HA/WT^ OHCs (Supplementary Tables 3 and 4).

Pairwise statistical tests (t-tests) are provided in Supplementary Table 3. To complete the statistical assessment, two-way ANOVAs were conducted for two hair cell types (IHCs and OHCs) and 5 genotypes (WT, α9^HA/WT^, α9^HA/HA^, α10^HA/WT^, α10^HA/HA^). Separate analyses were run to evaluate response amplitude, and response probability.

For response amplitude there was no interaction between genotype and hair cell type (F = 0.93, DFn = 4, DFd = 52, *p* = 0.4524). Genotype did impact amplitude (F = 3.24, DFn = 4, DFd = 52, *p* = 0.0189), however hair cell type (IHC vs. OHC) did not (F = 1.07, DFn = 1, DFd = 52, *p* = 0.305). This corresponds with the pairwise comparisons. The strongest effect was seen for IHCs of the α9^HA/HA^ genotype in which response amplitude was significantly smaller than in either the wild type or heterozygous condition (Supplementary Table 4).

For response probability the interaction of genotype and hair cell type was significant, F = 4.71, DFn = 4, DFd = 55, *p* = 0.0024. Further, the effect of genotype was highly significant, F = 5.81, DFn = 4, DFd = 55, *p* = 0.0006. Likewise, the effect of cell type was highly significant, F = 114.98, DFn = 1, DFd = 55, *p* < 0.001. That is, response probability was strongly affected both by hair cell type (OHC < IHC) and by genotype (homozygotes < heterozygotes or wildtype).

A quantitative analysis was made of efferent bouton volume per OHC in apical turns of adult WT and mutant mice. Reminiscent of the physiology data, this analysis revealed that bouton volume was significantly smaller in the homozygous mutant mice (α9^HA/HA^ 19.85 ± 8.87 and α10^HA/HA^ 19.98 ± 7.44) than in the WT (28.64 ± 9.05) or heterozygous mice (α9^HA/WT^ 28.38 ± 8.72 and α10^HA/WT^ 28.40 ± 8.56) (Supplementary Fig. 4).

## Discussion

Acetylcholine (ACh) was identified as the principal neurotransmitter released by the MOC efferents in the 1950s^55^, but the structure and molecular composition of ACh receptors in hair cells was largely unknown till α9α10 receptor subunits were cloned and assembled *in vitro*^15,56^ The presence and function of α9α10 receptors in IHCs and OHCs has been shown by mRNA expression, characterization of α9 and α10 knockout mice^15,57–59^, and physiology and pharmacology studies^39,60,61^. Still, the lack of reliable antibodies that identify α9 or α10 nAChRs has hampered the visualization of these receptors in the inner ear and other neuronal and non-neuronal tissues.

The use of CRISPR Cas9 technology to generate HA-tagged α9 and α10 nAChR knock-in mice provides for the first time an animal model to visualize their distribution in mouse tissue. This confirms the localization of HA-tagged α9 and α10 nAChR subunits in the prehearing IHCs, and in OHCs before and after the onset of hearing. HA-tagged nAChRs along the cochlear sensory epithelium aligned with presynaptic efferent terminals on OHCs. HA tagged immunopuncta, juxtaposed and post-synaptic to MOC nerve endings, reflect the expression of α9 and α10 proteins in hair cells. This deduction is compatible with previous physiological and histological findings that identified the cholinergic nature of cochlear efferent neurons in vertebrates and determined that the functional AChR of OHCs involves α9 and α10 subunits of the nicotinic AChR gene family^3,4,6–8,10,14,16,19,48^.

Although α9 subunits expressed alone in oocytes can form functional homomeric receptors, it is only when it is co-injected with the α10 subunit that it forms heteromeric receptors with a physiological and pharmacological profile like that of the native OHC nAChRs^15,16^. Thus, the native nAChR of hair cells is thought be a heteromer of both subunits. The coincident pattern of labeling for α9HA and α10HA proteins observed here supports this model. These two subunits may assemble into functional heteromeric receptors with distinct stoichiometries, (α9)_2_(α10)_3_ or (α9)_3_(α10)_2_^62^.

Previously the presence and function of α9 and α10 subunits in IHCs and OHCs has been shown by studying mRNA expression^15,57,58^, ACh induced efferent inhibition in IHC and OHC physiology^39,59,60^, and by α-bungarotoxin labeling^43,63^ in the prehearing cochlea. Earlier studies have shown that α9 and α10 nAChR mRNA are differentially regulated during cochlear maturation^37,38,56^. In mature IHCs α9 mRNA expression persists at pre-hearing levels, but α10 mRNA expression is down regulated. This down regulation of α10 mRNA coincided with the absence of ACh-mediated responses from IHCs at the onset of hearing when assessed by electrophysiological techniques^56,57,64^. This also coincides with the withdrawal of efferent contacts from IHCs and the maturation of MOC contacts onto OHCs^65^. HA-tagged α9 and HA-tagged α10 immunoclusters vanish from IHCs as the cochlear epithelium matures, showing that both proteins are down regulated. The discrepancy between α9 mRNA and protein expression could be attributed to post-translational modifications that influence protein expression, turnover or abundance. The present HA-tagged immunolabeling along with many functional studies show that persistent cytoplasmic mRNA does not necessarily translate into a functional protein. The transient postnatal appearance of HA-tagged nAChRs in IHCs further demonstrates the reliability of this method of visualization while illustrating that the modified gene products are translated and regulated like the native protein.

Efferent inputs onto immature IHCs are thought to be essential to the maturation and functional refinement of the afferent synapses of IHCs^66,67^. HA labeling showed that the immunoclusters of both α9 and α10 proteins in immature IHCs are abundant and small, consistent with earlier studies of efferent innervation^43,45,46,68,69^. In the post-hearing cochlea, α9α10 nAChRs form fewer and larger clusters in OHCs postsynaptic to MOC terminals.

It is also noteworthy that diffuse cytosolic HA immunolabeling was observed in developing as well as adult IHCs and OHCs. This diffuse labeling may reflect trapping of HA-tagged protein in endoplasmic compartments and recommends caution when interpreting somatic labeling in other tissues.

The present work describes efferent innervation, hearing, and cellular synaptic function in mice that were heterozygous or homozygous for the inserted HA tag. Hearing and efferent innervation were not different in the homozygous HA-tagged mice compared to heterozygous mice. Postsynaptic currents in all cell/genotypes included inward and outward components, consistent with the known participation of calcium-dependent potassium channels. Synaptic amplitudes, time course and response probability in the heterozygous mice were similar to values for wildtype mice. Postsynaptic amplitude and response probability were generally lower in the homozygous mice compared to heterozygotes. In line with physiology, the efferent boutons in the apical turn of homozygous mice were smaller than WT and heterozygous littermates, which may be related to smaller or less frequent vesicular release. It is also conceivable that multiple HA tags interfere with ligand-binding or gating to reduce postsynaptic response amplitude. This was seen most consistently where the HA tag was on the ligand-binding α9 subunit. Response amplitude of the homozygous α9^HA/HA^ IHCs was consistently smaller than in the heterozygote or the wildtype. (It is unclear why this was not observed for the OHCs). Still more speculative is why homozygous HA tags would impact presynaptic release, although a previous study identified nitric-oxide-dependent retrograde facilitation of efferent function^70^. If reduced postsynaptic responses in homozygous HA-tagged hair cells caused less calcium-dependent generation of NO, this in turn would reduce retrograde facilitation of efferent transmitter release. However, this cannot be the only explanation since postsynaptic response amplitude was not correlated with response probability in every case. While the underlying mechanisms remain to be determined, the possibility that HA homozygosity impacts both post- and presynaptic function suggests that heterozygous mice will be the better choice for future studies.

Alpha9 and/or α10 containing nAChRs have been detected by in situ hybridization in a multitude of neuronal and non-neuronal cell types. Examples include: the retina^71^, pituitary pars tuberalis and olfactory epithelium^15,56^, keratinocytes^72–76^, and cancer cells^77,78^. Antibody labeling has been shown for the native α9-containing nAChR in vestibular tissue^26^ or lymphocytes^20^. Also, α9 and α10 subunits are important targets in the treatment of neuropathic pain. Alpha9α10 nAChRs selective antagonists, like α-conotoxins (RgIA and Vc1.1) when administered subcutaneously or through intramuscular administration alleviate pain resulting from inflammatory or traumatic injury in chronic constriction nerve injury (CCI) and partial sciatic nerve ligation (PSNL) models of nerve injury^24^. The HA tagging of α9 and α10 nAChRs will open new avenues to study the distribution and function of nAChRs in different tissue types and mouse models of various pathologies in the future.

The present findings show that heterozygote HA-tagged α9 and HA-tagged α10 mice display normal hearing, typical efferent innervation, and display normal synaptic physiology similar to wild type mice. These mouse models offer a valuable tool that will help gain understanding into the role the efferent system in cochlear synaptic plasticity during development and aging, to understand its role in noise exposure, and will open new avenues to gain insight into cochlear function.

## Materials and methods

Animal use was performed according to protocols approved by Johns Hopkins Animal Care and Use Committee (ACUC). All experiments were performed in accordance with ACUC ethics guidelines and regulations. Mice from both sexes were used throughout our experiments and no significant differences were observed in the results from male and female mice. Each experimental finding has been reported by analyzing results from at least three different experiments with animals from across the mouse line colony.

### Generation of CRISPR knock-in HA-tagged α9 and α10 mice

CRISPR/ Cas9-gene editing^79^ was used to knock-in the HA tag nucleotides within exon 5 of either α9 or α10 nAChR subunits. Small guide RNA (sgRNA) and single stranded oligonucleotide constructs (ssDNA) containing the HA tag and spacer sequence for both α9 and α10 genes (Fig. 1; Supplementary Table 1) were synthesized by Integrated DNA Technologies (IDT, Coralville, Iowa). Small guide RNAs (sgRNA) to target the genomic DNA were selected according the criteria of lowest probability of off-target sites and optimal placement within a single exon and farthest from intron/exon junctions (Fig. 1). Also, the location of sgRNAs was selected within exon 5 in order to attach HA tag to the last amino acid of the C terminus of either protein. The homologous oligonucleotides inserted into exon 5 were 60 bp (α9) and 63 bp (α10) long. The insert was composed of a cassette containing a spacer sequence (33 bp for α9 and 36 bp for α10) linked at its 3′-end to the 27 bp HA tag oligonucleotide (Fig. 1; Supplementary Table 1). The methods used and construct design were adapted from Cunningham et al.^80^.

Pronuclear injection of one-cell embryos was performed by the JHU Transgenic Core. A mixture of Cas9 protein (30 ng/µl, PNABio), tracrRNA (0.6 µM, IDT), sgRNA (0.6 µM, IDT) and ssDNA oligo (10 ng/ul, IDT) diluted in RNAse-free TE buffer (10 mM Tris–HCl, pH 7.4, 0.25 mM EDTA) was injected into one cell B6SJL/F2 embryos (Jax labs). Micro-injected embryos were cultured for 24 h and implanted into the oviducts of pseudo pregnant ICR females (Envigo). All the above-mentioned procedures were performed as detailed in Nagy et al.^81^. Two to 3 weeks after birth tail snips were collected from the pups for screening. After genotyping and sequencing, results of potential carrier mice were analyzed for the anticipated in frame insertions. Mice with random insertions or deletions were euthanized. The mice with the desired knock-in insertion were kept and bred with FVB.129P2 wild type mice (Jax labs, strain # 004,828) and their offspring were genotyped and sequenced to confirm the germ-line transmission of the HA tag. Sequencing was done for the DNA construct and flanking regions of the insertion site. Both WT and knock in alleles were sequenced.

### Auditory brainstem responses (ABRs)

ABR procedures were based on work previously published^82,83^. Briefly, adult mice (6–10 weeks) were anesthetized with an intraperitoneal injection of 0.1 cc per 20 g body weight of a mixture of veterinary grade ketamine (100 mg/kg) and xylazine (20 mg/kg) in 14% ethyl alcohol. Anesthetized mice were placed on a regulated heating pad to maintain core body temperature within one degree of 37 °C. Sterile Petrolatum ophthalmic ointment was used to prevent corneal ulcers on the eyes of anesthetized mice. Three subdermal platinum electrodes were placed behind the left pinna (inverting) at the vertex of the head (non-inverting) and on the left side of the base of the tail (ground). Averaged ABR waveforms were generated from 300 repetitions of a click or pure-tone stimulus presented at a rate of 10 stimuli/second. Each stimulus was 5 ms in duration with a 0.5 ms rise and fall time. Stimuli were presented using a Fostex dome tweeter speaker (model FT28D) in a sound-attenuating chamber lined with acoustic foam. The ABR threshold was calculated by comparing the averaged peak-to-peak voltage during a 5 ms interval beginning 1 ms after the onset of the stimulus. The electrical noise level calculated from the averaged peak to peak voltage in a 5 ms window, 20 ms after the stimulus. The threshold was set as the stimulus level that produced a peak-to-peak response that was greater than 2 standard deviations above the electrical noise. The threshold value was generated by a custom Matlab-based software interfaced with Tucker Davis System Hardware based on the input–output function of ABR magnitude to stimulus level. For additional waveform analysis, a marker was placed at the initial baseline and at the positive and negative peaks of waves one to five. The latency was reported as the time from the stimulus to the peak of the first wave of the ABR waveform. This did not include the time that would cover the 30 cm from the speaker to the animal’s pinna. The wave 1 amplitude was calculated by subtracting the value of the first negative peak of the first wave of the ABR waveform from the first positive peak in microvolts. Graphs were generated using GraphPad Prism8.

### Immunohistochemistry

Temporal bones were collected from postnatal day 7–8 (P7–P8) and 3–8-week old adult mice by isoflurane anesthesia followed by decapitation. The temporal bones from adult mice were perfused with 4% paraformaldehyde (PFA) in 1× phosphate buffered saline (PBS) through the oval and round windows. P7–P8 mouse temporal bones were opened and cleared from surrounding connective tissue and immediately immersed in the same fixative. All samples were post-fixed in fresh fixative solution for 30–40 min at room temperature (RT), rinsed thoroughly with 1× PBS and micro dissected to tease out the coiled cochlear sensory epithelium. Reissner’s membrane and tectorial membrane were removed from the organ of Corti and the tissue was permeabilized in 1× PBS containing 10% normal donkey serum and 0.6% Triton x-100 for 1 to 2 h at RT. Primary antibodies mix was applied to the tissue samples and were incubated overnight at RT or at 4 °C. Following several rinses in 1× PBS, tissue samples were transferred to Alexa fluor conjugated secondary antibodies (Life technologies, 1:1000 dilution) solution supplemented with nuclear dye DAPI (1:5000) and were incubated for 1 to 2 h at RT. The samples were rinsed several times in 1× PBS and mounted in FluorSave antifade mounting medium (CalBiochem, San Diego, CA) using coverslips specific for confocal microscopy. Primary antibodies used in this study include monoclonal rabbit anti-HA (Cell Signaling Technologies, 1:100 #3724), monoclonal mouse anti-SV2 (Developmental Studies Hybridoma Bank-DSHB, 1:300), polyclonal goat anti-ChAT (Sigma-Millipore, 1:300), polyclonal chicken anti-neurofilament heavy chain (NFH) (Sigma-Millipore, 1:200).

Images were acquired on a LSM700 confocal microscope (Zeiss Axio Imager Z2) using 40× N.A. 1.30 and 63× N.A. 1.4. oil immersion objective and Images were processed using Fiji (RRID: SCR_002285)^84^, Photoshop CS6 (Adobe) and Illustrator CS6 (Adobe).

### Immunohistochemistry image analysis

Confocal stacks were loaded into the virtual reality software syGlass 1.5 (Istovisio, Inc., https://www.syglass.io/) for quantification of the number and volume of OHC efferent boutons. Heterozygous and homozygous α9HA and α10HA adult mice and wildtype controls were immunolabeled with DAPI, anti-ChAT and anti-HA antibodies to visualize the efferent boutons and HA labeling of the α9α10 nAChRs on OHCs. For each image analyzed, the efferent boutons under each OHC were counted using the Counting Tool in syGlass. First, all boutons were counted from all cells in an image with yellow counting dots. Then, all OHC nuclei were counted with green counting dots. The ratio of yellow to green counts was then graphed as shown in Supplementary Fig. 3. Images from the apical, middle and basal turns were included in the counting of efferent boutons per OHC. For quantification of the volume of efferent terminals in the apical turn, a region of interest was drawn around the entire OHC region of the image. Then, a mask was carved for the ChAT-labeled efferent bouton area under each OHC by hand. Each mask corresponded to the total efferent bouton volume under one OHC and was recorded in cubic microns (µm^3^) as derived from the voxel size of the confocal image.

### Electrophysiology

P9-11 mice were decapitated and organs of Corti were rapidly dissected in an extracellular saline containing (in mM): 144 NaCl, 5.8 KCl, 10 Hepes, 5 d-Glucose, 1.3 CaCl_2_, 0.9 MgCl_2_, and NaH_2_PO_4_, pH 7.4 (NaOH), 315–325 mOsm/Kg. Explants of apical organ of Corti were secured to the recording chamber under single strands of dental floss. Hair cells were visualized with Nomarski optics through a Zeiss Axioskop with a long working-distance 40× water immersion objective. Images were magnified 4× through a TV tube and viewed on a monitor through a CCD camera.

Whole-cell voltage-clamp recordings were made from inner or outer hair cells in the apical turn of freshly excised cochlear explants. Gigaohm-seal electrodes with a resistance of 3–5 MΩ were pulled from borosilicate glass capillaries and filled with an intracellular solution containing (in mM): 135 KCl, 10 Phosphocreatine-di-Tris, 5 EGTA, 5 Hepes, 2.5 MgATP, 0.5 Na_2_GTP, and 0.1 CaCl_2_, pH 7.2 (KOH), 295–305 mOsm/Kg. Recordings were made at room temperature (21–23 °C) in the same extracellular saline as used for dissection. Extracellular calcium was raised to 5 mM to increase release probability for some recordings. High potassium saline (40 mM) was made by substitution for sodium. Series resistances were compensated 40–70%, leaving an uncompensated series resistance usually below 9 MΩ. Efferent axons were stimulated by an electrically isolated constant current source (World Precision Instruments A360) through a 4–10 µm diameter monopolar stimulating electrode positioned about 20 µm modiolar to the recorded hair cell. Stimuli were 80 to 200 µsec long and ranged in amplitude from 150 µV to 3 mV. Stimuli were presented as 1 Hz trains.

Currents were recorded with pCLAMP10.7 software and an Axopatch 200B amplifier. Data were acquired at 25 kHz and low-pass filtered at 3 kHz after acquisition for analysis in Clampfit 10.7 software or MiniAnalysis (Synaptosoft). IPSCs were identified using a template search protocol in Clampfit 10.7 software or through the detection protocols in MiniAnalysis (Synaptosoft Inc. Decatur, GA.).

### Statistics

Pairwise comparisons were made by two-tailed t-test assuming unequal variance (Graphpad t-test calculator). Resulting *p*, t, and df values are reported in the text. Two-way ANOVAs were run for multiple comparisons of hair cell type and genotype, with outcome parameters reported in the text.

## Supplementary Information

Supplementary Information.

## Data Availability

Most of the data generated or analyzed during this study are included in this published article. All datasets from the current study are available from the corresponding author on request.
