# Supplementary material for: Characterization of HA-tagged α9 and α10 nAChRs in the mouse cochlea
Source: Sci Rep. 2020 Dec 11;10:21814. doi: 10.1038/s41598-020-78380-5 (PMC7733449; doi:10.1038/s41598-020-78380-5)
Supplement: Supplementary file 1 — Supplementary Information. [file 41598_2020_78380_MOESM1_ESM.pdf]

Characterization of HA-tagged α9 and α10 nAChRs in the mouse cochlea

Pankhuri Vyas, Megan Beers Wood, Yuanyuan Zhang[1], Adam C. Goldring[2], Fatima-Zahra Chakir, Paul Albert Fuchs, and Hakim Hiel*

The Center for Hearing and Balance, Otolaryngology-Head and Neck Surgery, Johns Hopkins University School of Medicine, Baltimore, MD, 21205, USA. 1. Permanent address: Otolaryngology-Head and Neck Surgery, Renmin Hospital of Wuhan University, Wuhan, 430060, Hubei, China. 2. Present address: Sutter Instrument Company, 1 Digital Drive, Novato, CA 94949.

*Corresponding author

Address for correspondence

Hakim Hiel
The Johns Hopkins University School of Medicine
Department of Otolaryngology Head and Neck Surgery
720 Rutland Avenue, Ross 818
Baltimore, MD 21205, USA
Tel: 410-955-3877
Fax: 443-287-4334
Email: hhiel1@jhmi.edu

Supplementary Table1. Sequence of constructs used in generating the CRISPR knock in α9HA and α10HA mice. * indicates introduced silent mutation site, XXX indicates PAM site, XXX indicates the Stop codon

Name	Size	Sequence
Hemagglutinin (HA) tag	27 bp	TACCCATACGATGTTCCAGATTACGCT
Spacer (linker)	33 bp<br>36 bp	AGCGGCGGCAGCGGCGGCCCGCCGGTGGCGACC (for Alpha9 construct)<br>GGCAGCGGAGGTAGCGGCGGTCCACCGGTGGCGACC (for Alpha10 construct)
α9/spacer/HA ssDNA construct	183 bp	ATGTGGATTTTCTTCGCTATGGTGTTTGTCATGA**CG*G**TCTTGATCATAGCAAGAGCAGATAGCGGCGGCAGCGGCGGCCCGCCGGTGGCGACCTACCCATACGATGTTCCAGATTACGCTTAGAAAGAAAGAGGAGGAGTGGGTTGGTAGGCATTCGATATTTGGAGAAAAAAAAATCAATAA
α9 sgRNA	20 bp	TCTTGATCATAGCAAGAGCA
α9 Forward primer P2	21 bp	GCACGCTATGAAGCACTGACA
α9 Reverse primer P1	25 bp	TGCTTGATTTAACCTGACACGCAGC
α10/spacer/HA ssDNA construct	200 bp	GAGTAATGGACCGCTTTTTCCTAGGCATCTTCTTCTGCATGGCTCTGGTCATGA**GT*C**TA*CTT*GTACTA*GTGCAAGCT*CTGGGCAGCGGAGGTAGCGGCGGTCCACCGGTGGCGACCTACCCATACGATGTTCCAGATTACGCTTAAGGGCCAGGAACTGGATTTCAGGGAGCTGTGATCACCTCAACACCACCAGGTGGG
α10 sgRNA	19 bp	CCTGGTACTGGTGCAAGCCC
α10 Forward primer P2	20 bp	ACGTAGCTTCCATTGCCGGC
α10 Reverse primer P1	20 bp	TGGGGAGAAACTGGCTAGTG

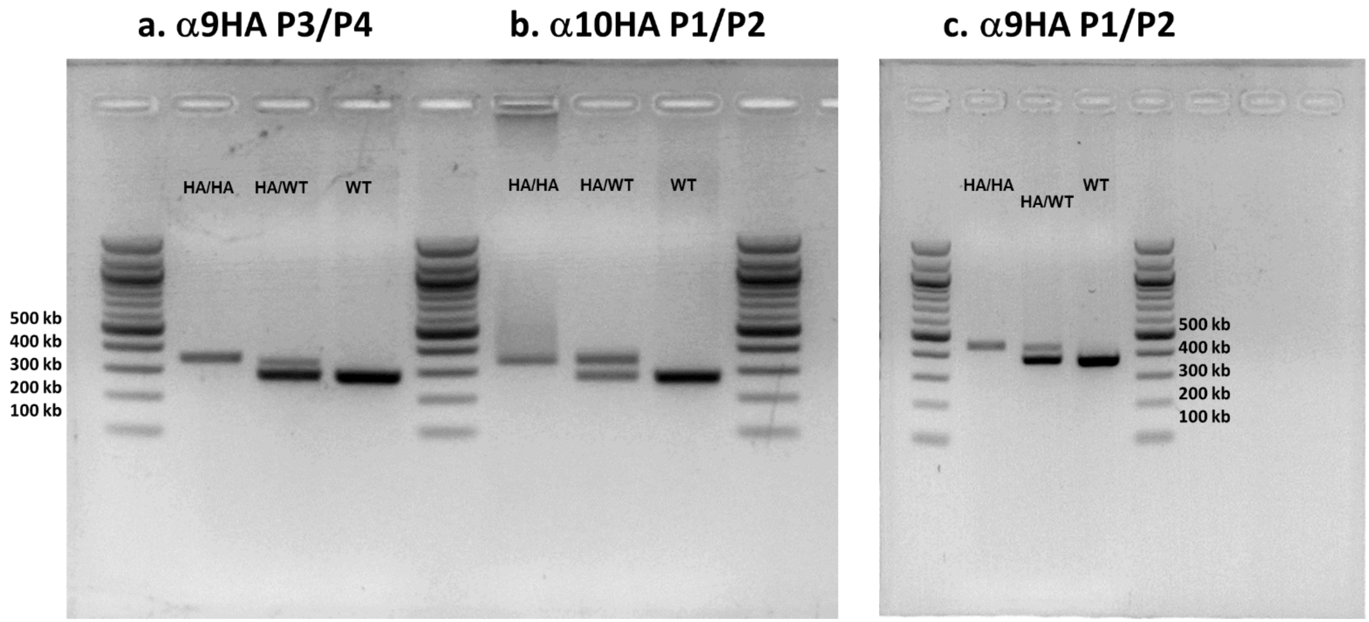

Supplementary Figure 1. Unprocessed PCR gels showing raw data for genotyping results of α9HA and α10HA mouse lines indicated in Fig.1. Alpha 9HA genotyping with primer pairs P3 and P4 (a), α10HA genotyping with primer pairs P1 and P2 (b), and α9HA genotyping with primer pairs P1 and P2 (c). P1 and P3 denote reverse primers, P2 and P4 denote forward primers, 100kb DNA ladder for size reference.

Only gel **b** and gel **c** were cropped and used to represent genotyping from α10HA and α9HA mice respectively in Figure 1 of the manuscript.

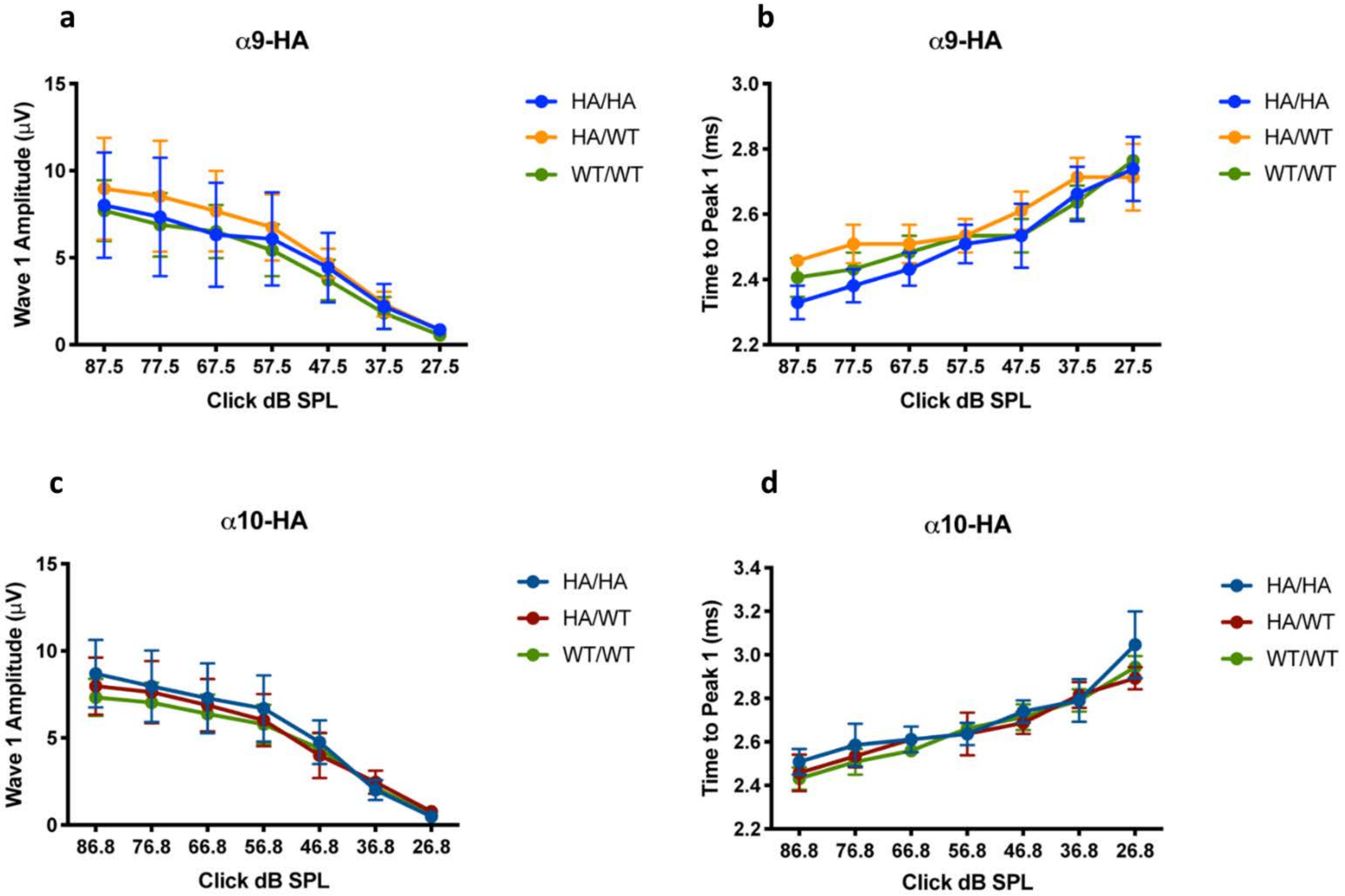

Supplementary Figure 2: Auditory Brainstem Responses peak amplitude and latency. Wave one amplitude (microvolts) was measured for the click stimulus from the maximum stimulation to threshold for all genotypes and associated wildtypes of α9HA (a) and α10HA mice (c). Latency (ms) was measured as the time from stimulus to the positive peak of wave one of the ABR waveform from the maximum stimulus level to threshold for the α9HA mice (b) and α10HA mice (d). A two-way ANOVA with Dunnett's correction was performed to compare each mutant genotype to the wildtype group. No significant effects were found for the mutant genotypes for wave one amplitude or latency to wave one when compared to wildtype. Each group consisted of 4 mice. These are the same animals analyzed in Figure 2. Error bars indicate standard deviation.

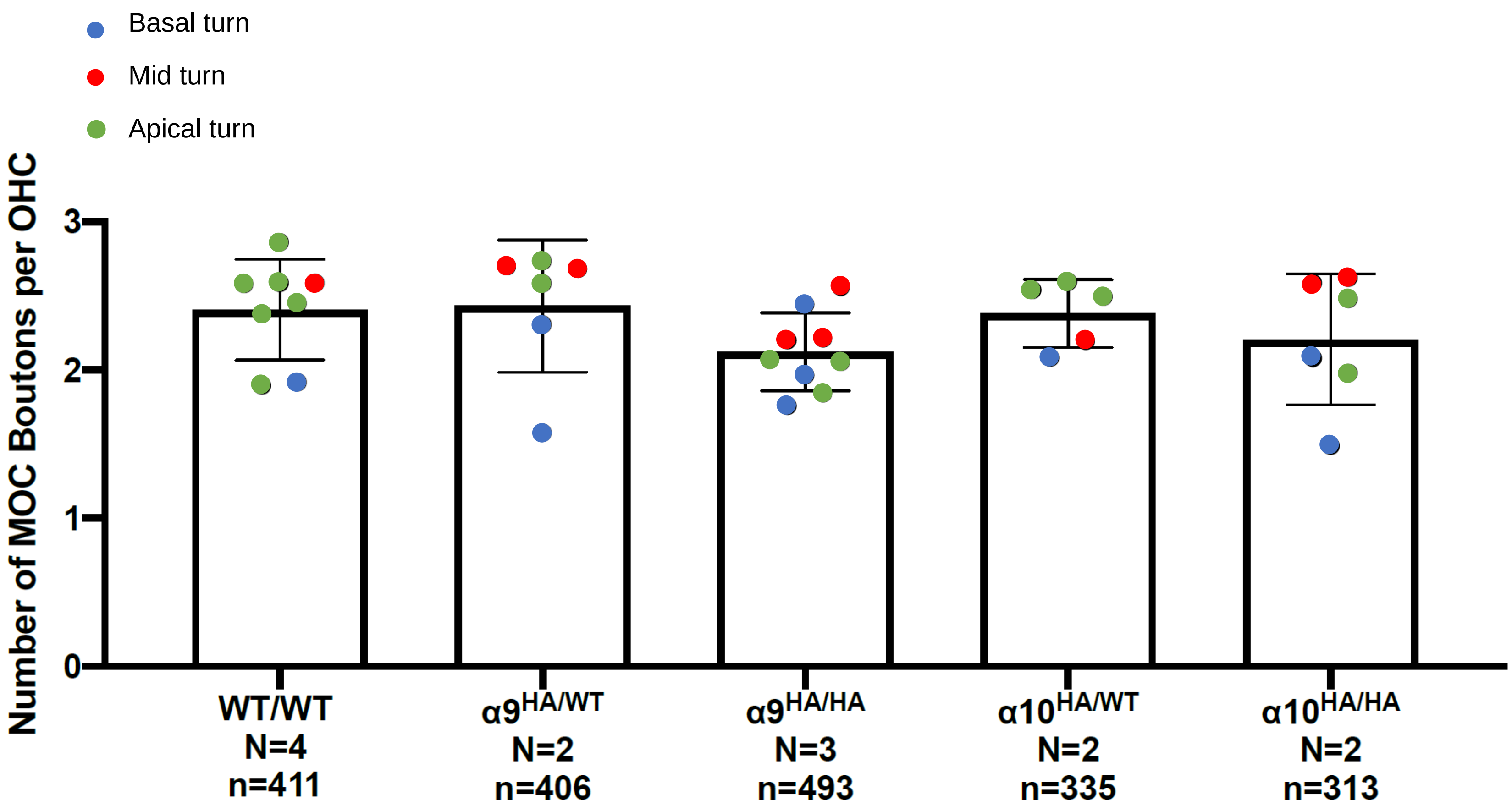

Supplementary Figure 3: Number of efferent boutons on OHCs was unaffected by HA-tagging of α9 or α10 subunits. Efferent boutons underneath OHCs were counted in the apical, middle and basal turns of the indicated genotypes. When the same turn from both ears was counted, the mean of that turn from that animal is shown. However, counts of each turn from each mouse is shown separately (Green-apical turn, Red-mid turn and Blue-basal turn). Unpaired student's t-tests compared each genotype to the wildtype data; and, no genotype was significantly different from the wildtype. $\alpha9^{HA/WT}$: p=0.91; $\alpha9^{HA/HA}$: p=0.07; $\alpha10^{HA/WT}$: p=0.88; $\alpha10^{HA/HA}$: p=0.35. N=number of animals. n=number of OHCs. Error bars indicate standard deviation.

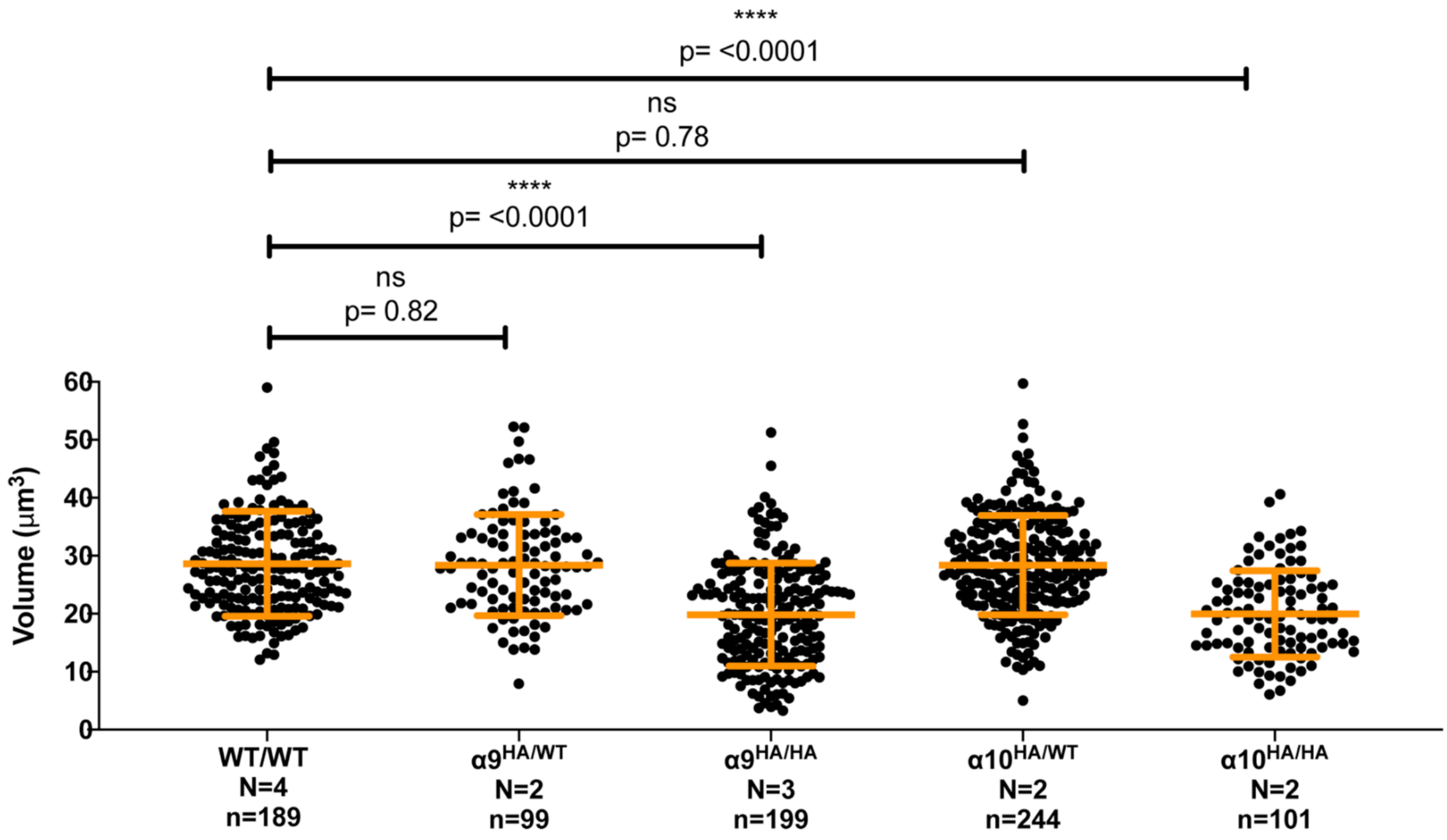

Supplementary Figure 4: Volume of the total efferent terminals on OHC is smaller in homozygous HA-tagged α9 and α10 mice. The volume (μm^3) of the ChAT-positive efferent bouton cluster below apical OHCs was quantified for α9 and α10 heterozygous and homozygous mice and their associated wildtypes. N= number of mice; n= number of OHCs analyzed. The p-values indicated are the result of unpaired student's t-tests comparing the indicated genotype to the wildtype data. Error bars indicate standard deviation.

Supplementary Table 2. Mean and standard deviation (SD) for amplitude, time constant of decay and response probability (p) of α9HA and α10HA mice (measured at -94 mV hair cell membrane potential).

**$\alpha9^{WT/HA}$**	**n = cells**	**N = mice**	**amp (pA)**	**tau (ms)**	**p response @ 1 Hz**	**ln(N/N0)**
IHC (1.3 Ca^{2+})	8	7	-37 ± 23	35 ± 3	0.72 ± 0.18	1.27
OHC (5.0 Ca^{2+})	4	3	-31 ± 9	33 ± 3	0.33 ± 0.21	0.4
**$\alpha9^{HA/HA}$**	**n = cells**	**N = mice**	**amp (pA)**	**tau (ms)**	**p response**	**ln(N/N0)**
IHC (1.3 Ca^{2+})	14	8	-16 ± 4	31 ± 3	0.37 ± 0.20	0.46
OHC (5.0 Ca^{2+})	5	4	-28 ± 19	32 ± 7	0.19 ± 0.05	0.21
**$\alpha10^{WT/HA}$**	**n = cells**	**N = mice**	**amp (pA)**	**tau (ms)**	**p response**	**ln(N/N0)**
IHC (1.3 Ca^{2+})	6	3	-39.5 ± 11.6	33 ± 11	0.85 ± 0.12	0.99
OHC (5.0 Ca^{2+})	6	5	-36 ± 8	35 ± 5	0.12 ± 0.09	0.13
**$\alpha10^{HA/HA}$**	**n = cells**	**N = mice**	**amp (pA)**	**tau (ms)**	**p response**	**ln(N/N0)**
IHC (1.3 Ca^{2+})	2	1	-15.5 ± 3.5	42.4 ± 10.5	0.51	
IHC (5.0 Ca^{2+})	5	4	-31 ± 15	41.2 ± 12.4	0.5 ± 0.1	0.78
OHC (5.0 Ca^{2+})	5	3	-27 ± 5	29 ± 6	0.06 ± .01	0.06
**WT/WT**	**n = cells**	**N = mice**	**amp (pA)**	**tau (ms)**	**p response**	**ln(N/N0)**
IHC (1.3 Ca^{2+})	8	7	-32.2 ± 17.3	53.4 ± 21.1	0.81 ± 0.27	1.66
OHC (5.0 Ca^{2+})	4	3	-38.3 ± 15.6	44.8 ± 7.4	0.17 ± 0.04	0.18

Supplementary Table 3. Pairwise comparison of amplitude and response probability by cell type and genotype in α9HA and α10HA mice.

IHCs				
**pairwise**	**Amplitude**	**t-test**	**Probability**	**t-test**
$\alpha9^{WT/HA}$	-37 ± 23	$p = 0.003$	0.72 ± 0.18	$p < 0.001$
$\alpha9^{HA/HA}$	-16 ± 4	$t = 3.388$	0.37 ± 0.20	$t = 4.087$
		df = 20		df = 20
$\alpha10^{WT/HA}$	-39.5 ± 11.6	$p = 0.033$	0.85 ± 0.12	$p < 0.001$
$\alpha10^{HA/HA}$	-15.5 ± 3.5	$t = 2.751$	0.5 ± 0.1	$t = 5.181$
		df = 6		df = 9
**OHCs**				
**pairwise**	**Amplitude**	**t-test**	**Probability**	**t-test**
$\alpha9^{WT/HA}$	-31 ± 9	$p = 0.782$	0.33 ± 0.21	$p = 0.025$
$\alpha9^{HA/HA}$	-28 ± 19	$t = 0.288$	0.19 ± 0.05	$t = 2.418$
		df = 7		df = 7
$\alpha10^{WT/HA}$	-35 ± 10	$p = 0.165$	0.12 ± 0.09	$p = 0.176$
$\alpha10^{HA/HA}$	-27 ± 5	$t = 1.519$	0.06 ± 0.01	$t = 1.469$
		df = 9		df = 9

Supplementary Table 4. Response probability IHC vs. OHC, by genotype in α9HA and α10HA mice. OHC response probability measured in 5 mM external calcium, IHCs in 1.3 mM calcium except for α10$^{HA/HA}$ IHCs. Two-tailed, unpaired t-test for all comparisons.

IHC vs OHC Response Probability								
**WT/WT**		**t-test**	**α9$^{WT/HA}$**			**α10$^{WT/HA}$**		
**IHC**	0.81 ± 0.27	p = 0.001	**IHC**	0.72 ± 0.18	p = 0.007	**IHC**	0.85 ± 0.12	p < 0.001
**OHC**	0.17 ± 0.04	t = 4.605	**OHC**	0.33 ± 0.21	t = 3.361	**OHC**	0.12 ± 0.09	t = 4.633
		df = 10			df = 10			df = 19
			**α9$^{HA/HA}$**			**α10$^{HA/HA}$**		
			**IHC**	0.37 ± 0.20	p = 0.046	**IHC**	0.54 ± 0.16	p < 0.001
			**OHC**	0.19 ± 0.05	t = 2.145	**OHC**	0.06 ± 0.01	t = 6.649
					df = 18			df = 10
